# Alzheimer's disease polygenic risk in early‐ and late‐onset Alzheimer's disease

**DOI:** 10.1002/alz.71066

**Published:** 2026-01-14

**Authors:** Julian V. Pentchev, Trever Jackson, Naazneen Khan, Thea J. Rosewood, Yen‐Ning Huang, Kwangsik Nho, Andrew J. Saykin, Ani Eloyan, Alexander Taurone, Maryanne Thangarajah, Meghan Riddle, Steven Salloway, Alireza Atri, Lawrence S. Honig, Erik C. B. Johnson, Raymond Scott Turner, Joseph C. Masdeu, Tatiana M. Foroud, David Clark, Dustin B. Hammers, Jeffrey L. Dage, Kala Kirby, Bernardino Ghetti, Kathy Newell, Chiadi U. Onyike, Gregory S. Day, Neill R. Graff‐Radford, Melissa E. Murray, David T. Jones, Clifford R. Jack, Prashanthi Vemuri, Alexandra Touroutoglou, Ranjan Duara, Ian Grant, Sharon Sha, Thomas S. Wingo, Laurel A. Beckett, Emily Rogalski, Mario F. Mendez, Joel Kramer, Renaud La Joie, Ganna Blazhenets, Lea T. Grinberg, Robert Koeppe, David A. Wolk, Paul Aisen, Rema Raman, Arthur Toga, Walter A. Kukull, Erik Musiek, Kyle B. Womack, Maria C. Carrillo, Gil D. Rabinovici, Bradford C. Dickerson, Liana G. Apostolova, Kelly N. H. Nudelman, for the Longitudinal Early‐Onset Alzheimer's Disease Study Consortium and the Alzheimer's Disease Neuroimaging Initiative

**Affiliations:** ^1^ Department of Neurology Indiana University School of Medicine Indianapolis USA; ^2^ Department of Medical and Molecular Genetics Indiana University School of Medicine Indianapolis USA; ^3^ Indiana Alzheimer's Disease Research Center Indiana University School of Medicine Indianapolis USA; ^4^ Department of Radiology and Imaging Sciences Indiana University School of Medicine Indianapolis USA; ^5^ Center for Statistical Sciences Brown University Providence USA; ^6^ Department of Neurology Alpert Medical School Brown University Providence USA; ^7^ Banner Sun Health Research Institute Sun City USA; ^8^ Department of Neurology Columbia University Irving Medical Center New York USA; ^9^ Department of Neurology and Human Genetics Emory University School of Medicine Atlanta USA; ^10^ Department of Neurology Georgetown University Washington D.C. USA; ^11^ Nantz National Alzheimer Center Houston Methodist and Weill Cornell Medicine Houston USA; ^12^ Department of Pathology and Laboratory Medicine Indiana University School of Medicine Indianapolis USA; ^13^ Department of Psychiatry and Behavioral Sciences Johns Hopkins University School of Medicine Baltimore USA; ^14^ Department of Neurology Mayo Clinic Jacksonville USA; ^15^ Department of Neuroscience Mayo Clinic Jacksonville USA; ^16^ Department of Neurology Mayo Clinic Rochester USA; ^17^ Department of Radiology Mayo Clinic Rochester USA; ^18^ Department of Neurology Massachusetts General Hospital and Harvard Medical School Boston USA; ^19^ Wien Center for Alzheimer's Disease and Memory Disorders Mount Sinai Medical Center Miami USA; ^20^ Department of Psychiatry and Behavioral Sciences Northwestern University Chicago USA; ^21^ Department of Neurology & Neurological Sciences Stanford University Palo Alto USA; ^22^ Department of Neurology University of California Davis USA; ^23^ Department of Public Health Sciences University of California Davis USA; ^24^ Department of Neurology Healthy Aging & Alzheimer's Research Care Center University of Chicago Chicago USA; ^25^ Department of Neurology University of California Los Angeles USA; ^26^ Department of Neurology University of California San Francisco USA; ^27^ Department of Pathology University of California San Francisco USA; ^28^ Department of Radiology University of Michigan Ann Arbor USA; ^29^ Department of Neurology Perelman School of Medicine University of Pennsylvania Philadelphia USA; ^30^ Alzheimer's Therapeutic Research Institute University of Southern California San Diego USA; ^31^ Laboratory of Neuro Imaging USC Stevens Neuroimaging and Informatics Institute Keck School of Medicine of USC Los Angeles USA; ^32^ Department of Epidemiology University of Washington Seattle USA; ^33^ Department of Neurology Washington University in St. Louis St. Louis USA; ^34^ Medical & Scientific Relations Division Alzheimer's Association Chicago USA

**Keywords:** Alzheimer's disease, early onset, genetics, polygenic

## Abstract

**INTRODUCTION:**

The genetic basis of sporadic early‐onset Alzheimer's disease (EOAD) remains largely unknown, prompting evaluation of late‐onset Alzheimer's disease (LOAD) polygenic risk in EOAD.

**METHODS:**

A LOAD polygenic score (PGS) was calculated in the Longitudinal Early‐onset Alzheimer's Disease Study (LEADS) and Alzheimer's Disease Neuroimaging Initiative (ADNI) study and tested for associations with AD risk, cognitive performance, and imaging and fluid biomarkers.

**RESULTS:**

Though PGS was elevated in LOAD and EOAD, it was not a significant predictor of EOAD adjusting for *APOE* ε4 carrier status and was not associated with age of EOAD onset (*p* = 0.106) or with cognitive performance (*p* = 0.417). In LEADS, greater LOAD PGS was associated with differences in neuroimaging and fluid biomarkers, including elevated synaptosomal‐associated protein 25 (SNAP‐25) (*p* = 2.3 × 10^−5^).

**DISCUSSION:**

While LOAD polygenic risk contributed minimally to EOAD onset and cognitive dysfunction, PGS association with fluid biomarkers in LEADS suggests a role for LOAD polygenic risk in EOAD pathophysiology.

**Highlights:**

- LOAD PGSs were elevated in both LOAD and EOAD compared to controls; however, LOAD PGS did not significantly predict EOAD risk, age at onset, or cognitive performance independent of *APOE* ε4 in the LEADS.
- Higher LOAD PGS was associated with lower amyloid PET Centiloids (less brain amyloid deposition) as well as lower CSF biomarker Aβ42 in LEADS (proxy marker suggesting higher brain amyloid deposition) in LEADS; these contradictory findings support the need for larger studies to further investigate whether LOAD PGS is associated with increased amyloid deposition in EOAD.
- Higher LOAD PGS was also associated with higher levels of CSF synaptosomal‐associated protein 25 (SNAP‐25), a key component of the SNARE complex, suggesting that LOAD genetic factors may contribute to dysregulation of synaptic transmission and/or pathological protein aggregation in EOAD.

## 1 BACKGROUND

Alzheimer's disease (AD) is a multifactorial neurodegenerative condition with both genetic and environmental contributors. To date, over 80 single nucleotide polymorphisms (SNPs) have been associated with increased AD risk.^1^ Similar to other diseases, polygenic scores (PGSs) have been developed to summarize the contribution of selected SNPs to AD risk. A PGS is generated from the results of a genome‐wide association study (GWAS); SNPs are selected from the GWAS results, often using a false discovery rate (FDR)‐ or Bonferroni‐corrected *p* value, and the effect size for each SNP. Within an individual in a target cohort, genotypes for each selected SNP are then weighted using the effect size from the GWAS results, and the resulting scores per SNP are summed or averaged to generate the final score.^2^, ^3^ This score is then used to assess each individual's genetic risk for a disease or trait, with higher scores typically indicating higher risk. These PGSs can then be analyzed to determine the cumulative contribution of the PGS to disease‐related endophenotypes, biomarkers, or other clinical measures, further enhancing the potential utility of the PGS for clinical risk prediction.

In AD, PGSs may explain additional disease risk beyond the apolipoprotein E (*APOE*) locus and have been associated with age of onset, disease progression, and clinical measures such as cognitive decline and biofluid and neuroimaging biomarkers.^4^, ^5^, ^6^, ^7^, ^8^, ^9^, ^10^ Given that only a small portion (∼10%) of early‐onset amyloid‐positive AD (EOAD, <65 years age of onset) is accounted for by identified pathogenic variants in the amyloid precursor protein (*APP*), presenilin 1 (*PSEN1*), or presenilin 2 (*PSEN2*) genes, a proportion of EOAD cases may be attributed to an extreme enrichment of late‐onset AD (LOAD, ≥65 years age of onset) genetic risk factors, leading to younger age of onset.^5^, ^11^ However, to date, there have been comparatively few studies investigating the contribution of LOAD genetic risk to EOAD with somewhat inconsistent consensus of the impact of LOAD genetic risk in EOAD.^4^, ^5^, ^6^, ^12^, ^13^, ^14^, ^15^, ^16^ Previous results from Cruchaga et al. indicated that in sporadic EOAD, an AD PGS had a higher odds ratio (OR) for case/control status than sporadic LOAD.^5^ Notably, however, a recent study by Mantyh et al. does not support this hypothesis.^6^ In this analysis, AD PGS and hazard scores were predictive of AD case/control status, but not age of onset in EOAD. Interestingly, they also showed that there was no significant difference in scores between EOAD and LOAD, even after selecting biomarker‐confirmed cases only. Given the significant association of AD PGS with case/control status, but not with age of onset, additional investigations are needed to clarify the role of LOAD polygenic risk in EOAD.

This study investigated the association of LOAD PGS in participants enrolled in the Longitudinal Early‐Onset Alzheimer's Disease Study (LEADS) cohort and the Alzheimer's Disease Neuroimaging Initiative (ADNI). PGSs were compared across cohorts to replicate results from Mantyh et al. in a larger EOAD cohort and to assess the associations between LOAD PGS and cognitive, neuroimaging, and fluid biomarkers of EOAD.^6^

**RESEARCH IN CONTEXT**

1. **Systematic review**: The authors searched PubMed for primary studies and reviews of PGS analyses in EOAD and LOAD. While LOAD PGS is widely studied and associated with LOAD risk, few studies have explored its relationship to EOAD risk and biomarkers.
2. **Interpretation**: Our findings show that LOAD PGS does not predict EOAD risk or age of onset and is not associated with cognitive performance. However, LOAD PGS is associated with Centiloid values of amyloid PET and CSF Aβ42/40 and synaptosome‐associated protein 25 (SNAP‐25). This suggests a role in EOAD pathophysiology despite limited predictive value for EOAD clinical onset.
3. **Future directions**: Future research in larger cohorts is needed to develop and validate PGS for EOAD and to investigate the biological mechanisms linking PGS to AD‐related biomarker changes. Integrating multi‐omic data and larger, more diverse cohorts will enhance understanding of the genetic architecture and pathophysiology of EOAD.

## 2 METHODS

### 2.1 Ethics statement

The LEADS and ADNI studies were both conducted in accordance with the 1975 Declaration of Helsinki, the Good Clinical Practice guidelines, and regulations for the protection of human subjects research. Written informed consent was obtained from participants in both cohorts. Protocols employed aligned with the Health Insurance Portability and Accountability Act, the International Conference on Harmonization, and all relevant state and federal regulations and were approved by Institutional Review Boards.

### 2.2 LEADS cohort

LEADS is an international, multisite, prospective observational study enrolling participants with a clinical diagnosis of EOAD (age 40 to 64 years) at enrollment and cognitively normal (CN) similarly aged controls. The goal of LEADS is specifically to enroll and follow sporadic EOAD, rather than familial EOAD. This cohort and protocols have been extensively described in previous publications.^17^, ^18^, ^19^, ^20^, ^21^, ^22^, ^23^, ^24^, ^25^, ^26^ Briefly, affected patients are screened to enroll those without multiple first‐degree relatives with EOAD to reduce the likelihood of enrolling individuals with pathogenic variants in autosomal‐dominant AD genes. Enrolled participants undergo amyloid positron emission tomography (PET) imaging to identify EOAD and amyloid‐negative cognitively impaired participants (EOnonAD). Affected participants complete genetic screening to identify individuals carrying AD‐causing variants in *APP*, *PSEN1*, and *PSEN2*. Given that some cases, though clinically presenting as AD, were amyloid‐negative, affected participants were also screened for pathogenic variants in progranulin (*GRN*) and microtubule‐associated tau protein (*MAPT*) genes, as well as for pathogenic repeat expansions in the *C9ORF72* gene. Carriers wishing to receive results were excluded from the study; carriers not wishing to receive results were followed but were excluded from analysis. LEADS participants receive standard clinical assessments, including medical and family history, medications, and clinical examinations. Cognitive assessments include the National Alzheimer's Coordinating Center (NACC) Uniform Data Set (UDS) version 3.0, as well as the Mini‐Mental State Examination (MMSE), Montreal Cognitive Assessment (MoCA), Rey Auditory Verbal Learning Test (RAVLT), and the Alzheimer's Disease Assessment Scale‐Cognitive Subscale (ADAS‐Cog).^27^, ^28^, ^29^, ^30^, ^31^, ^32^ Visits also include a blood draw, other cognitive and functional measurements, and additional brain imaging.^17^

Genotyping for LEADS participants was performed in three batches at the Children's Hospital of Philadelphia using the Illumina Global Screening Array (GSA) version 2.0 (Illumina, Inc., San Diego, CA, USA). Standard quality control procedures for GWAS data were performed with PLINK versions 1.9 and 2.0, including filtering for genotype missingness, minor allele frequency (MAF), subject missingness, and Hardy–Weinberg equilibrium (HWE).^33^, ^34^, ^35^ Genotype‐inferred sex was checked against reported sex, and GWAS data were compared with genotype data generated in‐house at the National Centralized Repository for Alzheimer's Disease and Related Dementias (NCRAD; https://www.ncrad.org/) using a 96‐SNP custom microfluidic genotype array to confirm sample identity. GENESIS, SNPRelate, and GWASTools R libraries (R version 4.0.4) were used to create an identity‐by‐descent (IBD) matrix, based on the KING method of momentum, which accounts for within‐ and between‐family relationships differently, to pass into the PCAir function for the production of the PCA eigenvectors.^36^, ^37^, ^38^, ^39^, ^40^ Data were imputed with the Michigan Imputation Server 2 using the 1000 Genomes Phase 3 data and phasing with Eagle.^41^, ^42^ Additional quality control was performed to filter out rare variants with *R* ^2^ < 0.3 and common variants with *R* ^2^ < 0.5, as well as SNPs not in HWE, and SNPs with MAF < 0.01. Non‐Hispanic European‐ancestry LEADS participants were identified by genetic clustering of principal components (PCs) 1–3 using 1000 Genomes Phase 3 data and k‐means clustering with sklearn.cluster in Python version 3.11.^43^, ^44^ *APOE* genotype data were obtained separately from the NCRAD for LEADS participants.

### 2.3 ADNI cohort

ADNI is a multiphase, multisite longitudinal study of mild cognitive impairment and AD that has been described extensively in previous publications.^45^, ^46^, ^47^, ^48^, ^49^, ^50^, ^51^, ^52^, ^53^, ^54^ Briefly, participants are enrolled at over 60 sites in the United States and Canada to study the progression of AD from normal aging to mild cognitive impairment and AD. ADNI participants have extensive longitudinal sample collections and data available, including demographics and clinical assessments and questionnaires, neuropsychological testing, multimodal neuroimaging, and subsets of participants with GWAS, genomics, and other omics data sets.

Genotyping for ADNI participants was performed using the Illumina Human 610‐Quad BeadChip, Human OmniExpress Beadchip, Omni 2.5M platform, or the Global Screening Array BeadChip (Illumina, Inc., San Diego, CA, USA). Standard quality control procedures for GWAS data were performed. Imputation was done for each data platform separately using the Haplotype Reference Consortium Panel r1.1, as described previously.^7^ Non‐Hispanic European‐ancestry ADNI participants were identified by genetic clustering using HapMap 3 genotype data and multidimensional scaling.^55^

### 2.4 PGS calculation

The PGS used for this analysis was obtained from the Polygenic Score Catalog (https://www.pgscatalog.org). PGS000026, generated by Desikan et al., was used to select SNPs and weights for the calculation (Table S1).^56^, ^57^ Of the 33 SNPs included in the original score, three were not present in the GWAS data (Table S2). LDlink LDproxy (https://ldlink.nih.gov/?tab = ldproxy) was used to pull variants in linkage disequilibrium with these three SNPs, using the 1000 Genomes CEU reference population with a 500‐bp window.^58^ From these results, the SNP in highest linkage disequilibrium (D’ and *R* ^2^) with the original SNP and present in the GWAS data was selected as a proxy. No proxy SNP was identified for rs115124923 on chromosome 6 within the GWAS data, so this SNP was not included in the final score calculation, which used the remaining 32 variants. PGS was calculated using PLINK version 2.0, using a summed score centered on zero. Missing individual genotype data were imputed using the allele frequency in the data. PGSs were calculated separately for LEADS and ADNI, both using the same method. Once the scores were imported into the combined data set, a *z*‐score PGS (PGS_z_) with the calculation (PGS − overall mean PGS)/overall standard deviation (SD) PGS. PGS_z_ was used in logistic regressions and receiver operating characteristic (ROC) assessment of classification, to enable comparisons of odds ratios with *APOE* ε4 carrier status on a similar scale.

### 2.5 Participant selection

**FIGURE 1:**
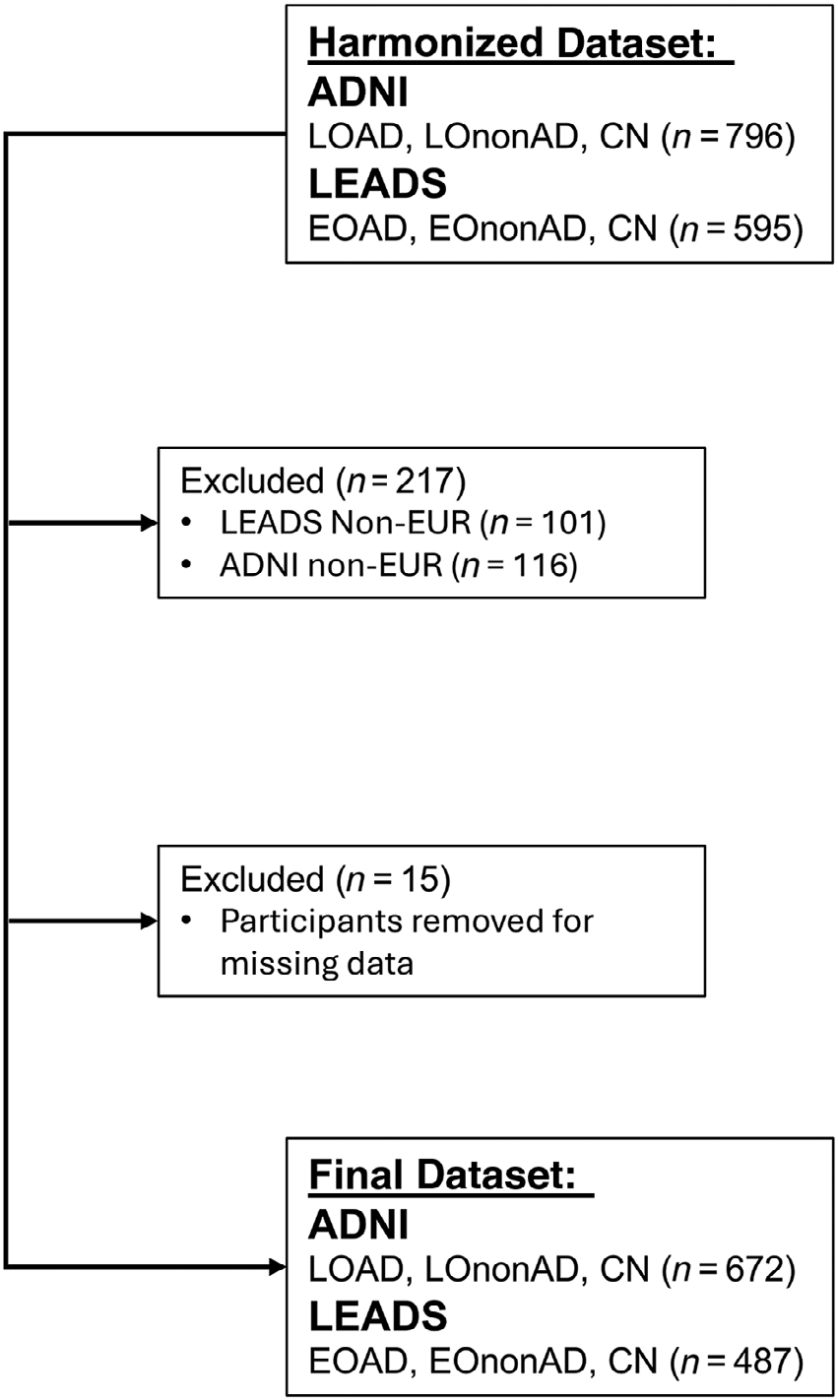
Study subject selection. Harmonized data including demographic, clinical, and cognitive data were obtained for the Alzheimer's Disease Neuroimaging Initiative (ADNI) (*n* = 796) and Longitudinal Early‐onset Alzheimer's Disease Study (LEADS) (*n* = 595). Within this initial selection, individuals with non‐European ancestry were excluded (*n* = 232) to ensure the polygenic score was directly applicable to the target population. Fifteen additional participants were removed due to missing data, leaving a final sample size of 1159 for analysis.

The LEADS Data Management and Statistics Core and Neuroimaging Cores assembled a harmonized dataset using LEADS criteria to assess amyloid‐positive/negative status, to allow for direct comparisons of LEADS and ADNI data.^17^ This harmonized dataset included 595 LEADS participants and 796 ADNI participants, classified as EOAD/EOnonAD/CN (LEADS) or LOAD/late‐onset amyloid‐negative cognitively impaired (LOnonAD)/CN. ADNI cases were only included if age of onset was >65 years, and additional ADNI data including demographic, clinical, and cognitive data were obtained for this cohort from the Laboratory for Neuroimaging (www.loni.usc.edu). Of these 1391 ADNI and LEADS participants, 487 LEADS participants and 672 ADNI participants with European ancestry had genetic and cognitive data available for analysis (Figure 1) and were included in the final dataset. Given the sensitivity of the PGS to differences in genetic ancestry, and the relatively small numbers of participants who had non‐European genetic ancestry in both cohorts, we limited the present analysis to only individuals with European genetic ancestry.

### 2.6 Cognitive assessments

Cognitive domain calculations in LEADS and ADNI have been described by Hammers et al.^21^ MMSE and MoCA scores were obtained for individuals in both datasets and compared for missingness. MMSE was selected for follow‐up testing as the cognitive test with the fewest missing scores. MMSE scores were pre‐adjusted for years of education using linear regression, and the unstandardized residual of this analysis was used to test for within‐cohort differences in adjusted MMSE by PGS. Additionally, within the LEADS cohort EOAD participants (*n* = 330), cognitive domain data for episodic memory, language, processing speed and attention, visuospatial skills, and working memory were assessed for mean differences by PGS tertiles. Each domain was pre‐adjusted within LEADS participants by linear regression with baseline age, sex, education, and global cognition (MMSE; see Hammers et al. for additional information).^21^

### 2.7 Amyloid PET imaging

For both ADNI and LEADS, previous work generated standardized quantified ^18^F‐Florbetaben or ^18^F‐Florbetapir tracer uptake values from amyloid PET neuroimaging data for a subset of both cohorts.^26^ As described in Lagarde et al., a composite neocortical standardized uptake value ratio quantification was extracted from a magnetic resonance imaging‐based processing pipeline, using mean activity in the FreeSurfer‐parcellated whole cerebellum as the reference region, and then converted to Centiloids.^26^, ^59^

Of the 487 LEADS participants included in our analysis, 380 of them had Centiloid data; similarly, 485 ADNI participants had Centiloid data. Given that amyloid PET was used in the classification of diagnostic groups, Centiloid values are collinear with diagnosis. Thus, rather than testing for prediction or classification of diagnosis using these variables, Centiloid data were tested for Pearson's correlation with PGS_z_ separately within each study for all study participants. Post hoc analysis was performed within LOAD in ADNI and within diagnostic groups in LEADS.

### 2.8 LEADS biofluid biomarkers

A subset of LEADS participants (*n* = 174) consented to the optional lumbar puncture, as described in Dage et al., and cerebrospinal fluid (CSF) biomarkers were measured for amyloid beta (Aβ) 42 and 40, tau phosphorylated at threonine 181 (pTau181), total Tau (tTau), neurofilament light chain (NfL), neurogranin (Ng), visinin‐like protein 1 (VILIP‐1), YKL‐40, and synaptosome‐associated protein 25 (SNAP‐25).^19^

To investigate the association between PGS and CSF biomarkers, 171 participants with PGS and CSF biomarker data were analyzed using linear regression after standardizing the data. Given the skewness in biomarker distributions, log transformation was applied to normalize CSF biomarker levels, followed by *z*‐score standardization to facilitate comparability across different biomarkers and diagnostic groups. The dataset was stratified into three diagnostic groups: CN (*n* = 38), EOAD (*n* = 99), and EOnonAD (*n* = 34). To visually assess the relationship between PGS and the synaptic biomarker SNAP‐25, scatter plots with group‐specific regression lines were generated using the ggplot2 library in R. The linear regression model (lm function) was applied within each group to quantify the strength and direction of the association between PGS and biomarker expression. The *p* value for PGS, extracted from the regression model, provided statistical significance for the observed associations, with a threshold of *p* < 0.05 considered significant. As several of the tested biomarkers had some missing data, final cohort numbers included in significant analyses are indicated in the results.

### 2.9 Statistical analysis

This study utilized IBM SPSS Statistics, version 27.0.1.0, 64‐bit edition for all analyses unless otherwise indicated. Clinical and demographic variables were assessed for differences by diagnostic group using one‐way ANOVA or chi‐squared analyses. Binary logistic regressions were performed for case (EOAD and/or LOAD)/control status, covarying for sex and PGS_z_, with and without *APOE* ε4 carrier status. *P* values reported are unadjusted, but Bonferroni‐corrected *p* value threshold is also indicated in the results tables. Computed probabilities were used to run ROC to visualize classification. A tertile PGS variable was created and used as a categorical variable (1 to 3, corresponding to low, medium, and high PGS values) for Cox regression and Kaplan–Meier survival analysis with age of AD onset, and as a grouping factor for non‐parametric analyses of differences in adjusted MMSE (described in Section 2.6). Adjusted cognitive scores were assessed for differences by PGS tertiles in LEADS EOAD using Kruskal–Wallis tests, with Mann‐Whitney U pairwise comparisons performed for significant results.

## 3 RESULTS

### 3.1 Demographics

The demographic characteristics of participants across the six groups in the combined dataset are summarized in Table 1 below. The CN group included both ADNI and LEADS participants. As expected, significant differences were observed in age, with LOAD and LOnonAD groups being older (mean = 77.5, SD = 5.8 and mean = 78.0, SD = 6.3, respectively, *p* < 0.001) compared to other groups. Years of education were highest in the CN group (mean = 16.9, SD = 2.2, *p* < 0.001). The proportion of females varied significantly, with CN group having the highest representation (56.9%, *p* < 0.001). More *APOE* ε4 carriers were present in the LOAD group (65.0%, *p* < 0.001) compared to all other groups, while the EOAD group had more carriers (53.3%) compared to EOnonAD (30.4%) and CN (32.6%). Cognitive performance, measured by MMSE score, was highest in the CN group (mean = 29.1, SD = 1.2, *p* < 0.001) as expected, with lower scores observed in the other groups, particularly EOAD (mean = 21.5, SD = 5.3, *p* < 0.001). PGS means were higher in LOAD and EOAD (mean = 0.0061, SD = 0.0159, and mean = 0.0007, SD = 0.0155, respectively, *p* < 0.001), while controls and amyloid‐negative cases had negative PGS means.

**TABLE 1:** Combined cohort demographics.

Variable	CN (*n* = 350)	EOAD (*n* = 330)	EOnonAD (*n* = 92)	LOAD (*n* = 277)	LOnonAD (*n* = 110)	Statistic^*^ (*p* value)
Mean age (SD)	70.5 (9.7)	58.7 (2.9)	58.5 (5.7)	77.5 (5.8)	78.0 (6.3)	395.3 (<0.001)
Mean education (SD)	16.9 (2.2)	15.7 (2.4)	15.7 (2.8)	16.0 (2.7)	16.4 (2.5)	12.7 (<0.001)
Count (%) female	199 (56.9%)	171 (51.8%)	36 (42.4%)	114 (41.2%)	37 (33.6%)	30.1 (<0.001)
Mean age of onset (SD)	–	55.6 (4.1)	55.1 (5.9)	73.9 (6.1)	74.2 (6.7)	523.7 (<0.001)
Count (%) *APOE* ε4 carrier	114 (32.6%)	176 (53.3%)	28 (30.4%)	180 (65.0%)	14 (12.7%)	130.1 (<0.001)
Mean MMSE(SD)	29.1 (1.2)	21.5 (5.3)	26.1 (3.8)	25.2 (3.3)	27.3 (2.6)	203.8 (<0.001)
Mean PGS (SD)	−0.0045 (0.0139)	0.0007 (0.0155)	−0.0055 (0.0140)	0.0061 (0.0159)	−0.0089 (0.0118)	32.286 (<0.001)
Mean PGS (SD)	−0.0045 (0.0139)	0.0007 (0.0155)	−0.0055 (0.0140)	0.0061 (0.0159)	−0.0089 (0.0118)	32.286 (<0.001)

Abbreviations: CN, cognitively normal; EOAD, early‐onset Alzheimer's disease; EOnonAD, early onset non‐Alzheimer's disease; LOAD, late‐onset Alzheimer's disease; LOnonAD, late onset non‐Alzheimer's disease; PGS, polygenic score; SD, standard deviation.

* Statistics are either *F* value (*p* value) for one‐way ANOVAs with continuous variables or *χ^2^* (*p* value) for nominal and categorical variables.

### 3.2 PGS_z_ comparison

**FIGURE 2:**
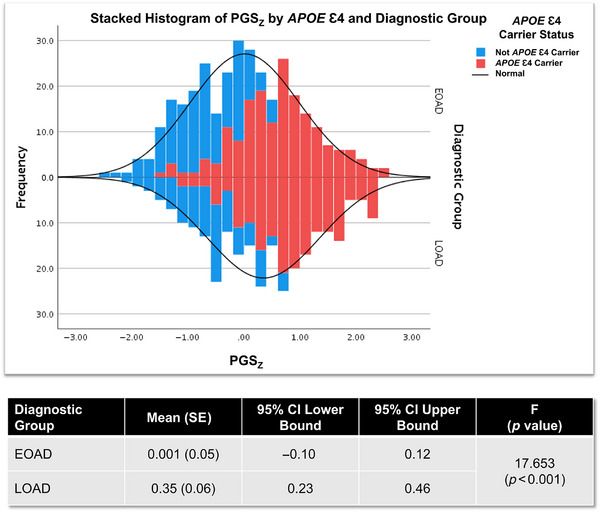
Distribution of *z*‐score polygenic scores (PGS_z_) in Alzheimer's disease. Histograms were generated for PGS_z_ frequency in early‐onset Alzheimer's disease (EOAD) in the Longitudinal Early‐onset Alzheimer's Disease Study (LEADS) and late‐onset AD (LOAD) in the Alzheimer's Disease Neuroimaging Initiative (ADNI). Blue bars represent *APOE* ε4 non‐carriers, while red bars represent apolipoprotein E (*APOE*) ε4 carriers. Group means with standard errors (SE), 95% confidence intervals (CIs) lower and upper bounds, and the *F* statistic and *p* value for EOAD versus LOAD PGS_z_ comparison are shown in the table.

Differences in PGS_z_ means were assessed using a one‐way ANOVA, comparing EOAD and LOAD groups. As shown in Figure 2, mean PGS_z_ was significantly higher in LOAD cases (mean = 0.35, 95% confidence interval [CI] 0.23 to 0.46) than in EOAD cases (mean = 0.01, 95% CI −0.1 to 0.12).

### 3.3 AD predictors

**FIGURE 3:**
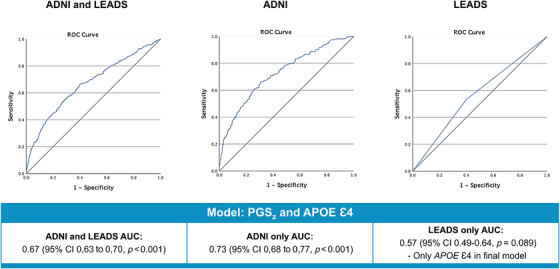
Area under the curve (AUC) for genetic predictors of Alzheimer's disease (AD). *Z*‐score polygenic score (PGS_z_), apolipoprotein E (*APOE*) ε4 carrier status, and sex were modeled as predictors of AD in the combined cohort, in the Alzheimer's Disease Neuroimaging Initiative (ADNI) only, and in the Longitudinal Early‐onset Alzheimer's Disease Study (LEADS) only. Graphs show the receiver operating characteristic (ROC) curves in blue for each model, with sensitivity on the *y*‐axis and specificity on the *x*‐axis, and the random classifier line is shown in black. Model AUC is indicated under each graph, with the 95% confidence intervals (CIs) lower and upper bounds and unadjusted *p* value. Results below the multiple testing correction (Bonferroni‐adjusted *p* < 0.0167) are indicated by an asterisk.

The associations between sex and PGS_z_, with and without *APOE* ε4, and AD case/control status were evaluated with binary logistic regression. Results from these analyses are shown in Table 2. In the combined cohort including sex and PGS_z_, the model was statistically significant (*χ^2^* [2] = 62.73, *p* < 0.001), although it explained only a modest amount of variance (Nagelkerke *R* ^2 ^= 8.7%) and correctly classified only 63.1% of participants as AD or CN. PGS_z_ was a significant predictor of AD case/control status (OR = 1.697, *p* < 0.001). The model, including both PGS_z_ and *APOE* ε4, in essence separating out the effects of *APOE* ε4 carrier status and the remainder of the PGS_z_ signal, was also significant (*χ^2^* [3] = 77.26, *p* < 0.001), explaining 10.6% of variance and correctly classifying 64.4% of participants as AD or CN. Assessment of the ROC for this model in the combined cohort showed an AUC of 0.67 (*p* < 0.001, Figure 3).

**TABLE 2:** Logistic regressions for case/control status.

Cohort	Model	*χ^2^* (*p* value) [*R* ^2^]	Percentage correct CN/AD^*^	Predictor	B	Exp(B)	Wald	*df*	*p* value
ADNI and LEADS (*n* = 957)	PGS_z_	62.73 (< 0.001) [8.7%]	20.9%/87.5%	Sex	−0.39	0.68	7.78	1	0.005^**^
				PGS_z_	0.53	1.70	49.77	1	<0.001^**^
	*APOE* ε4 and PGS_z_	77.26 (< 0.001) [10.6%]	31.1%/83.5%	Sex	−0.39	0.68	7.73	1	0.005^**^
				*APOE* ε4	0.72	2.05	14.31	1	<0.001^**^
				PGS_z_	0.28	1.32	7.80	1	0.005^**^
ADNI only (*n* = 562)	PGS_z_	81.10 (< 0.001) [17.9%]	68.8%/59.9%	Sex	−0.63	0.53	12.02	1	<0.001^**^
				PGS_z_	0.769	2.16	59.03	1	<0.001^**^
	*APOE* ε4 and PGS_z_	93.20 (< 0.001) [20.4%]	69.5%/65.7%	Sex	−0.64	0.53	11.87	1	<0.001^**^
				*APOE* ε4	0.83	2.29	11.92	1	<0.001^**^
				PGS_z_	0.48	1.62	13.90	1	<0.001^**^
LEADS only (*n* = 395)	PGS_z_	3.63 (0.057) [1.5%]	0.0%/100.0%	PGS_z_	0.28	1.32	3.54	1	0.060
	*APOE* ε4 and PGS_z_	3.90 (0.05) [1.7%]	0.0%/100.0%	*APOE* ε4	0.54	1.71	3.81	1	0.050
ADNI and LEADS cases only (*n* = 607)	PGS_z_	23.52 (< 0.001) [5.1%]	41.5%/74.8%	Sex	0.41	1.51	6.09	1	0.014
				PGS_z_	−0.34	0.71	16.09	1	<0.001^**^

Abbreviations: ADNI; Alzheimer's Disease Neuroimaging Initiative; APOE, apolipoprotein E; LEADS, Longitudinal Early‐onset Alzheimer's Disease Study; PGS_z_, *Z*‐score polygenic score.

* For ADNI and LEADS cases‐only comparison, % classification indicates percentage correctly classified as EOAD or LOAD, respectively.

** Result below Bonferroni‐corrected *p* value significance threshold *p* = 0.007 (0.05/7 models).

Post hoc analysis of within‐cohort models was performed to investigate PGS_z_ and *APOE* ε4 as predictors of LOAD or EOAD separately. In the ADNI cohort, as expected, PGS_z_ and *APOE* ε4 were significant predictors of LOAD (PGS_z_ OR = 1.62, *p* < 0.001 and *APOE* ε4 OR = 2.29, *p* < 0.001), with the model explaining 20.4% of the variance (*χ^2^* [3] = 93.20, *p* < 0.001). In the LEADS cohort, the model with PGS_z_ showed a trend for predicting increased risk of EOAD (OR = 1.32, *p* = 0.060). However, assessment of the model including both PGS_z_ and *APOE* ε4 showed that PGS_z_ was not associated with EOAD independent of *APOE* in the LEADS cohort, with the final model only including *APOE* ε4 (OR 1.71, *p* = 0.05). This model also only explained 1.7% of the variance in the data (*χ^2^* [1] = 3.90, *p* = 0.05). Results for within‐cohort logistic regression results are shown in Table 2, with ROCs shown in Figure 3.

A logistic regression model of PGS_z_ and sex as predictors of EOAD compared to LOAD was significant (*χ^2^* [2] = 23.52, *p* < 0.001); however, PGS_z_ had an OR < 1 (OR = 0.71, *p* < 0.001), indicating that individuals with higher PGS_z_ were more likely to be LOAD rather than EOAD. Replication of this analysis in the subset of EOAD (*n* = 140) and LOAD (*n* = 87) cases with *APOE* ε3/ε3 genotypes showed that PGS_z_ was not a significant predictor of EOAD and had a similar OR of 0.78 (*p* = 0.262).

### 3.4 PGS and age of onset

**FIGURE 4:**
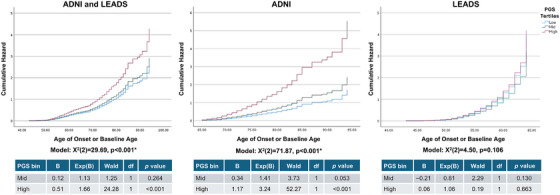
Polygenic score (PGS) risk for Alzheimer's disease (AD) by age. PGS tertiles (low‐risk/blue, mid‐risk/green, and high‐risk/red) were assessed for cumulative hazard for AD by age in the combined cohort, in the Alzheimer's Disease Neuroimaging Initiative (ADNI), and in Longitudinal Early‐onset Alzheimer's Disease Study (LEADS) using Cox regressions. Below each graph, model significance is shown (*indicates *p* values below the Bonferroni‐corrected *p* value threshold of *p* < 0.0167), as well as tables with statistics for mid‐ and high‐risk groups compared to the low‐risk group.

PGS was binned into ordinal tertiles and assessed for differences in age of AD onset (combined cohort), LOAD, or EOAD (Figure 4). While in the overall model (*χ^2^* [2] = 29.69, *p* < 0.001) and the ADNI‐only model (*χ^2^* [2] = 71.87, *p* < 0.001), the high‐risk group was associated with greater hazard of AD, in the LEADS cohort this relationship was not observed (*χ^2^* [2] = 4.50, *p* = 0.106). In ADNI, there was nearly 10 years’ difference in mean age of AD onset between the high‐risk group (mean 74.69, 95% CI 73.67 to 75.72) compared to the low‐risk group (mean 83.27, 95% CI 81.38 to 85.17). Younger mean age of onset in the high‐risk PGS group was also observed within a post hoc analysis stratified by *APOE* ε4 carrier status in both carriers and non‐carriers (Tables S3 and S4). However, in LEADS, age of onset was not significantly different between PGS risk groups.

### 3.5 PGS and cognition

Education‐adjusted MMSE (MMSE_adj_) was assessed for mean differences by PGS tertiles within each cohort separately using Kruskal–Wallis non‐parametric tests. In the ADNI cohort, MMSE_adj_ was significantly different across PGS tertile groups (*H*[2] = 17.04, *p* < 0.001), and pairwise comparisons showed the high‐risk PGS group MMSE_adj_ mean was significantly lower compared to the low‐risk group (*U* = 68.98, Bonferroni‐corrected *p* < 0.001). In contrast, in LEADS, MMSE_adj_ distributions were not significantly different between PGS tertile groups (*H*[2] = 1.75, *p* = 0.417).

Within the LEADS EOAD group, exploratory analyses were also performed to assess differences across five cognitive domains between PGS risk groups. Table S5 shows the results of the linear regression models used to adjust cognitive domain scores. Non‐parametric analysis of pre‐adjusted cognitive domains in EOAD participants did not identify any differences between PGS risk groups surviving multiple testing correction (Table 3), though a trend for differences in processing speed and attention (*p* = 0.017, pairwise comparisons shown in Table S6) and visuospatial skills (*p* = 0.072) was observed.

**TABLE 3:** Adjusted cognitive domains by polygenic score (PGS) tertiles within late‐onset Alzheimer's disease (LOAD) participants.

Adjusted cognitive domain	Test statistic	Degrees of freedom	*P* value^*^
Episodic memory	0.30	2	0.863
Language	4.29	2	0.118
Processing speed and attention	8.16	2	0.017
Visuospatial skills	5.25	2	0.072
Working memory	4.24	2	0.120

* No results pass Bonferroni‐adjusted *p* value cut‐off of *p* < 0.01.

### 3.6 PGS and amyloid PET centiloids

Within both ADNI and LEADS, harmonized amyloid PET Centiloids were assessed for correlation with PGS_z_. In ADNI, Centiloids were positively correlated with PGS_z_ for all participants (*r*[485] = 0.350, *p* < 0.001). Within LOAD participants (*r*[131] = 0.107, *p* = 0.224), a trend for positive correlation was observed, though this did not reach statistical significance. In LEADS, Centiloids showed a trend for positive correlation with PGS_z_ (*r*[380] = 0.094, *p* = 0.069), though this did not reach statistical significance. Post hoc analysis within diagnostic groups showed that while CN participants’ Centiloid values were positively correlated with PGS_z_ (*r*[60] = 0.281, *p* = 0.029), EOnonAD participants’ Centiloid values were not significantly correlated with PGS_z_ (*r*[68] = 0.044, *p* = 0.720), while in the EOAD group, centiloid values were negatively correlated with PGS_z_ (*r*[252] = –0.177, *p* = 0.005).

### 3.7 PGS and CSF biomarkers

**FIGURE 5:**
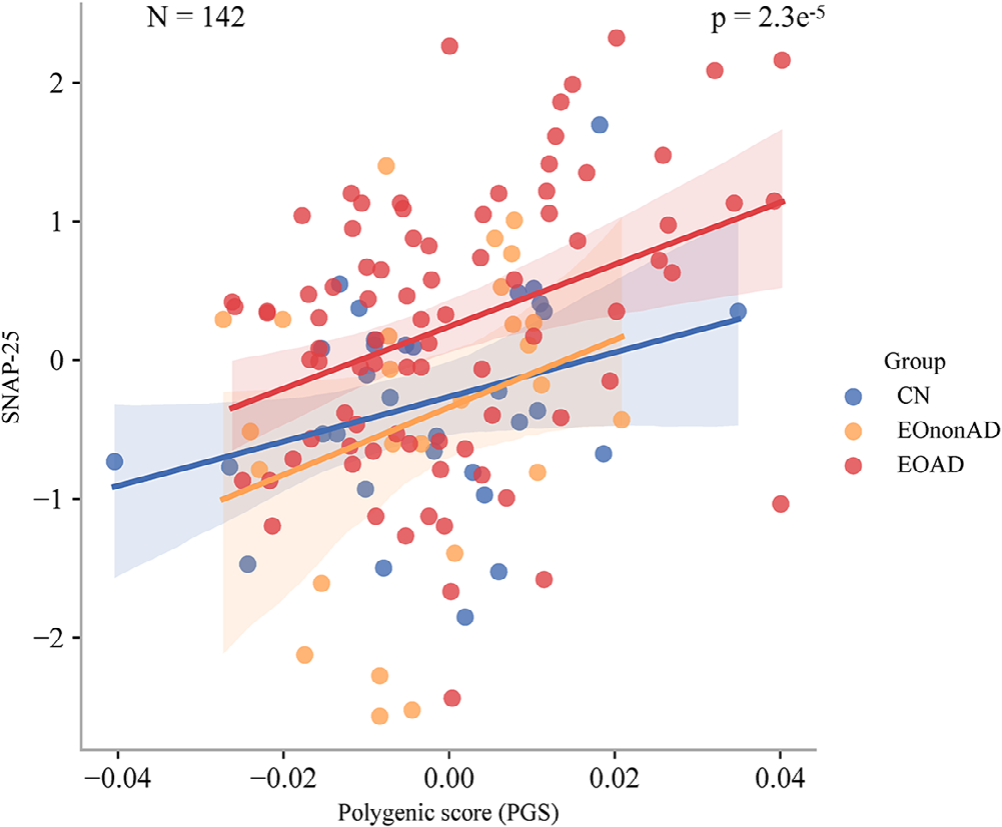
Polygenic score (PGS) association with cerebrospinal fluid (CSF) biomarker synaptosome‐associated protein 25 (SNAP‐25). This graph depicts the association of PGS (*x*‐axis) with SNAP‐25 measured in CSF (*y*‐axis) in the Longitudinal Early‐onset Alzheimer's Disease Study (LEADS) (*n* = 142 with CSF biomarker data). Each point represents a participant, and points, along with fit lines, are color coded (controls [CN] in blue, early‐onset Alzheimer's disease [EOAD] participants in red, and early‐onset amyloid‐negative cognitively impaired participants (EOnonAD) in orange. This association was significant (*p* = 2.3 × 10^−5^), passing the Bonferroni‐adjusted *p* value cut‐off for multiple testing of *p* < 0.006.

Within the subset of individuals with CSF biomarker data in LEADS (*n* = 171) stratified by diagnostic group, PGS was tested for associations with Aβ 42/40, pTau181, tTau, NfL, Ng, VILIP‐1, YKL‐40, and SNAP‐25. While a nominally significant association of higher PGS with lower CSF Aβ 42/40 was observed (*n* = 169, uncorrected *p* = 0.03), the only other biomarker to show a significant association was SNAP‐25. Intriguingly, higher PGS were associated with higher levels of CSF SNP‐25 (*n* = 142, *p* = 2.3 × 10^−5^), as shown in Figure 5. This result persisted after correction for multiple testing of CSF biomarkers (*p* < 0.006).

## 4 DISCUSSION

We compared the impact of a LOAD PGS on clinical profiles in EOAD and LOAD, as well as the impact of LOAD PGS on CSF biomarkers in EOAD. While we observed higher PGS in both LOAD and EOAD, and while PGS was a significant predictor of AD in the combined cohort as well as in ADNI, in LEADS, PGS was not a significant predictor of EOAD independent of *APOE* ε4 carrier status. Furthermore, while higher PGS were associated, as expected, with earlier age at onset and poorer global cognition (MMSE) in ADNI, these associations were absent in LEADS. Additionally, PGS tertiles were not associated with significant differences in specific cognitive domains in LEADS EOAD after multiple testing corrections. These results add to growing evidence that rather than EOAD resulting from a higher burden of LOAD genetic risk, EOAD appears to be genetically distinct from LOAD and likely driven by different genetic etiology, including high‐risk rare variants or distinct polygenic risk. These findings align with emerging evidence that other risk factors beyond LOAD genetics may contribute to EOAD risk and progression. In particular, recent work has implicated blood–brain barrier disruption and vascular–metabolic factors as potential contributors to EOAD pathogenesis.^60^ Integrating vascular and metabolic markers with genomic profiles may therefore be critical to more fully characterize EOAD heterogeneity.

Assessment of AD biomarkers CSF Aβ42/40 and amyloid PET neuroimaging in LEADS showed conflicting results. LEADS participants with higher LOAD polygenic risk have CSF profiles indicating more AD‐like pathology, with lower Aβ42/40 compared to EOnonAD and controls.^61^ However, analysis of amyloid deposition in the brain measured by Centiloids shows that LEADS EOAD participants with higher polygenic AD risk apparently have less amyloid deposition than those with lower risk. Given that all EOAD participants were categorized based on amyloid PET neuroimaging as amyloid‐positive, while we might expect a difference in the effect size of the relationship of amyloid and AD PGS between CSF and PET, we did not expect to observe a difference in the direction of effect between the two modalities.^62^ This unexpected observation, including more participants than the CSF analysis, highlights the importance of further investigation of the relationship of amyloid accumulation and polygenic AD risk in EOAD. We postulate that the relationship of LOAD polygenic risk with amyloid accumulation may be confounded by as‐yet unidentified genetic factors driving EOAD‐related pathological processes, but follow‐up is required with larger cohorts to further clarify the role of LOAD polygenic risk in amyloid accumulation in EOAD.

Interestingly, we also observed that individuals with higher polygenic AD risk also have elevated CSF SNAP‐25. SNAP‐25 is a target soluble N‐ethylmaleimide‐sensitive factor (NSF) attachment protein receptor (t‐SNARE), a key component of the SNARE complex, and plays a vital role in synaptic transmission.^63^ Dysregulation of this protein has been linked with impaired synaptic plasticity and neuronal communication, as well as potentially contributing to pathological protein aggregation in neurodegenerative disease.^64^ These findings may reflect greater pathological burden in higher PGS individuals, despite preserved or compensatory cognitive mechanisms. Alternatively, they may suggest that polygenic risk from LOAD studies tags a specific biological subtype within EOAD with unique phenotypic features. Additional biomarkers – such as α‐synuclein, glial fibrillary acidic protein, and soluble TREM2 – could help delineate glial and synaptic mechanisms underlying polygenic risk effects. While these data were unavailable for this cohort, integrating such markers in future EOAD studies will be essential to clarify how LOAD‐derived genetic risk translates into molecular and cellular pathology.

The association between higher PGS and increased SNAP‐25 further supports the biological validity of the PGS, even within EOAD, and is consistent with previous work linking PGS to synaptic and neuronal injury markers. That this association survived multiple testing correction underscores the potential of polygenic risk to inform mechanistic differences, even when not predictive of clinical onset in EOAD. Our findings shed additional light on the role of LOAD polygenic risk in EOAD. While Mantyh et al. suggested a modest predictive utility of PGS in EOAD, our results suggest a more nuanced view: PGS may not predict EOAD per se but may still be biologically informative and may be helpful in subtyping sporadic EOAD.

This study was subject to several limitations. While our analysis includes a larger sample size of sporadic EOAD than many previous reports, sample size is still a limiting factor, particularly in terms of the number of comparisons performed to more fully assess PGS associations with cognitive performance and fluid biomarkers. While we adjusted for multiple testing for cognitive domains and for fluid biomarkers, it will be important for larger studies to show replication of these results. It is also possible that more subtle effects of LOAD PGS would be detectable in a larger EOAD cohort; it will be important for future studies to validate these findings in larger studies. Additionally, this study is focused on individuals with European genetic ancestry, as we were not powered to assess PGS in other genetic ancestries in LEADS and ADNI. It will be vital for future studies to assess LOAD PGS targeted to other genetic ancestral groups within the context of EOAD, to understand if these findings translate across populations. Finally, this study is focused on assessing LOAD PGS in EOAD; however, it is entirely possible that sporadic EOAD individuals have genetic risk factors not captured by LOAD PGS. Larger studies are required to assess and replicate polygenic risk in sporadic EOAD.

In summary, our findings highlight the complex genetic architecture of sporadic EOAD. While LOAD polygenic risk contributes meaningfully to disease risk and progression in LOAD, its role in EOAD appears to be more nuanced, as it is less predictive of clinical outcomes, but may be biologically informative via association with fluid biomarkers. This suggests that LOAD PGS may aid in characterizing EOAD subtypes and pathological pathways, underscoring the heterogeneity within EOAD and the need for refined genetic models tailored specifically to EOAD. Future studies leveraging larger, more diverse cohorts with clinical, genetic, and molecular data will be critical to fully delineate the genetic landscape of EOAD and to translate these insights into improved diagnostics and personalized therapeutic strategies.

## CONFLICT OF INTEREST STATEMENT

Dr. Andrew J. Saykin receives support from multiple NIH grants (P30 AG010133, P30 AG072976, R01 AG019771, R01 AG057739, U19 AG024904, R01 LM013463, R01 AG068193, R01 AG092591, T32 AG071444, U01 AG068057, U01 AG072177, U19 AG074879, as well as U24 AG074855). He has also received in‐kind support from Avid Radiopharmaceuticals, a subsidiary of Eli Lilly (PET tracer precursor) Gates Ventures, LLC, and Sanofi (proteomics panel assays on IADRC and KBASE participants as part of the Global Neurodegeneration Proteomics Consortium), and has participated in scientific advisory boards (Bayer Oncology, Eisai, Novo Nordisk, and Siemens Medical Solutions USA, Inc.) and an Observational Study Monitoring Board (MESA and NIH/NHLBI), as well as External Advisory Committees for multiple NIA grants. He also serves as Editor‐in‐Chief of *Brain Imaging and Behavior*, a *Springer Nature Journal*. Jeffrey L. Dage is an inventor on patents or patent applications assigned to Eli Lilly and Company relating to the assays, methods, reagents, and/or compositions of matter for p‐tau assays and Aβ targeting therapeutics. Jeffrey L. Dage has/is served/serving as a consultant or on advisory boards for Eisai, AbbVie, Genotix Biotechnologies Inc., Gates Ventures, Gate Neurosciences, Dolby Family Ventures, Karuna Therapeutics, Alzheimer's Disease Drug Discovery Foundation, AlzPath Inc., Cognito Therapeutics, Inc., Eli Lilly and Company, Prevail Therapeutics, Neurogen Biomarking, Spear Bio, Rush University, University of Kentucky, Tymora Analytical Operations, MindImmune Therapeutics, Inc., Early is Good, and Quanterix. Jeffrey L. Dage has received research support from ADx Neurosciences, Fujirebio, Roche Diagnostics and Eli Lilly, and Company in the past 2 years. Jeffrey L. Dage has received speaker fees from Eli Lilly and Company and LabCorp. Jeffrey L. Dage is a founder and advisor for Monument Biosciences and Dage Scientific LLC. Jeffrey L. Dage has stock or stock options in Eli Lilly and Company, Genotix Biotechnologies, MindImmune Therapeutics Inc., AlzPath Inc., Neurogen Biomarking, and Monument Biosciences. Dr. Gregory S. Day reports no competing interests directly relevant to this work. His research is supported by NIH (R01AG089380, U01AG057195, U01NS120901, U19AG032438, and P30AG062677). He serves as a consultant for Arialys Therapeutics and as a topic editor (Dementia) for DynaMed (EBSCO). He is a co‐project PI for a clinical trial in anti‐NMDAR encephalitis, which receives support from NIH/NINDS (U01NS120901) and Amgen Pharmaceuticals. He has developed educational materials for Continuing Education Inc. and Ionis Pharmaceuticals. He owns stock in ANI Pharmaceuticals. Dr. Gregory S. Day's institution received in‐kind contributions for radiotracer precursors for tau PET neuroimaging in studies of memory and aging (via Avid Radiopharmaceuticals, a wholly owned subsidiary of Eli Lilly). Dr. Gil D. Rabinovici received research support from Avid Radiopharmaceuticals, GE Healthcare, Life Molecular Imaging, and Genentech. He has served as a paid consultant for Alector, Bristol Myers Squibb, C2N, Eli Lilly, Johnson & Johnson, Merck, Roche, and Novo Norodisk. He is Associate Editor for *JAMA*. Dr. Bradford C. Dickerson has served as a consultant for Acadia, Alector, Arkuda, Biogen, Cervomed, Eisai, Ilios, Lantheus, Lilly, Merck, Novo Nordisk, and Quanterix, He receives royalties from Cambridge University Press, Elsevier, and Oxford University Press. Dr. Liana G. Apostolova research is supported by grants or contracts from NIH/NIA, the Alzheimer's Association, Avid Radiopharmaceuticals, Life Molecular Imaging, Roche Diagnostics, and Eli Lilly. She has received consulting fees or honoraria from Biogen, Prothena, IQVIA, Genetech, Siemens, Corium, Eli Lilly, GE Healthcare, Eisai, Roche Diagnostics, Alnylam, Otsuka, AAN, MillerMed, NACC CME, CME Institute, MJH Physician Education Resource, WebMD, PeerView, Weil Cornell, and MedLearning Group. She serves on boards for IQVIA, NIA R01 AG061111, UAB Nathan Schock Center, New Mexico Exploratory ADRC, and FDA. She has leadership or fiduciary roles in Medical Science Council Alzheimer's Association Greater Indiana Chapter, Alzheimer's Association Science Program Committee, FDA PCNS Advisory Committee, and the Beeson Program Committee. The remaining authors have no conflicts of interest to disclose.

## CONSENT STATEMENT

Written informed consent was obtained from all participants in both the LEADS and ADNI cohorts.

## Supporting information



Supporting Information

Supporting Information

## Data Availability

The LEADS Data Sharing Policy employs the principles of Productivity, Transparency, Fairness, and Inclusiveness (https://leads‐study.medicine.iu.edu/researchers). Data requests may be submitted to the Data Sharing Committee for review. Requests that do not overlap with ongoing or planned investigations of LEADS principal and co‐investigators will be reviewed for scientific merit, feasibility, and appropriateness of the applicant's qualifications and resources, as well as Institutional Review Boards approval for data use. If approved, de‐identified data will be made available through the Laboratory of NeuroImaging (LONI; https://loni.usc.edu). Similarly, all ADNI data utilized in this analysis are accessible through LONI to approved investigators (https://adni.loni.usc.edu/data‐samples/adni‐data/#AccessData).
