# Supplementary material for: Alzheimer's disease polygenic risk in early‐ and late‐onset Alzheimer's disease
Source: Alzheimers Dement. 2026 Jan 14;22(1):e71066. doi: 10.1002/alz.71066 (PMC12802089; doi:10.1002/alz.71066)
Supplement: Supplementary file 1 — Supporting Information [file ALZ-22-e71066-s002.docx]

**Supplementary Tables**

**Table S1. PGS SNPs**

| **rsID** | **Chr** | **Position (BP)** | **Effect Allele** | **Other Allele** | **Effect Weight** | **Locus Name** | **Haplotype** |
| --- | --- | --- | --- | --- | --- | --- | --- |
| rs7412 | 19 | 45412079 | APOE e2 | APOE e3 | -0.47 | APOE | TRUE |
| rs429358 | 19 | 45411941 | APOE e4 | APOE e3 | 1.03 | APOE | TRUE |
| rs4266886 | 1 | 207685786 | C | T | -0.09 | CR1 | FALSE |
| rs61822977 | 1 | 207796065 | A | G | -0.08 | CR1 | FALSE |
| rs6733839 | 2 | 127892810 | C | T | -0.15 | BIN1 | FALSE |
| rs10202748 | 2 | 234003117 | A | C | -0.06 | INPP5D | FALSE |
| rs115124923 | 6 | 32510482 | C | A | 0.17 | HLA-DRB5 | FALSE |
| rs115675626 | 6 | 32669833 | G | A | -0.11 | HLA-DQB1 | FALSE |
| rs1109581 | 6 | 47678182 | C | T | -0.07 | GPR115 | FALSE |
| rs17265593 | 7 | 37619922 | T | C | -0.23 | BC043356 | FALSE |
| rs2597283 | 7 | 37690507 | A | C | 0.28 | BC043356 | FALSE |
| rs1476679 | 7 | 100004446 | T | C | 0.11 | ZCWPW1 | FALSE |
| rs78571833 | 7 | 143122924 | A | C | 0.14 | AL833583 | FALSE |
| rs12679874 | 8 | 27230819 | A | G | -0.09 | PTK2B | FALSE |
| rs2741342 | 8 | 27330096 | C | T | 0.09 | CHRNA2 | FALSE |
| rs7831810 | 8 | 27430506 | G | A | 0.09 | CLU | FALSE |
| rs1532277 | 8 | 27466181 | C | T | 0.21 | CLU | FALSE |
| rs9331888 | 8 | 27468862 | G | C | 0.16 | CLU | FALSE |
| rs7920721 | 10 | 11720308 | A | G | -0.07 | CR595071 | FALSE |
| rs3740688 | 11 | 47380340 | T | G | 0.07 | SPI1 | FALSE |
| rs7116190 | 11 | 59964992 | G | A | 0.08 | MS4A6A | FALSE |
| rs526904 | 11 | 85811364 | T | C | -0.2 | PICALM | FALSE |
| rs543293 | 11 | 85820077 | G | A | 0.3 | PICALM | FALSE |
| rs11218343 | 11 | 121435587 | T | C | 0.18 | SORL1 | FALSE |
| rs6572869 | 14 | 53353454 | G | A | -0.11 | FERMT2 | FALSE |
| rs12590273 | 14 | 92934120 | T | C | 0.1 | SLC24A4 | FALSE |
| rs7145100 | 14 | 107160690 | G | C | 0.08 | abParts | FALSE |
| rs74615166 | 15 | 64725490 | T | C | -0.23 | TRIP4 | FALSE |
| rs2526378 | 17 | 56404349 | A | G | 0.09 | BZRAP1 | FALSE |
| rs117481827 | 19 | 1021627 | T | C | -0.09 | C19orf6 | FALSE |
| rs7408475 | 19 | 1050130 | G | C | 0.18 | ABCA7 | FALSE |
| rs3752246 | 19 | 1056492 | C | G | -0.25 | ABCA7 | FALSE |
| rs7274581 | 20 | 55018260 | T | C | 0.1 | CASS4 | FALSE |

rsID = dbSNP ID; Chr = chromosome, BP = base pair

**Table S2. Proxy SNPs for PGS000026**

| **Original rsID** | **Proxy rsID** | **Chr*** | **Position (BP)** | **Effect Allele** | **Other Allele** |
| --- | --- | --- | --- | --- | --- |
| rs4266886 | rs4562624 | 1 | 207685965 | C | T |
| rs115124923 | N/A* |  |  |  |  |
| rs1109581 | rs6900318 | 6 | 47675297 | G | C |

rsID = dbSNP ID; Chr = chromosome, BP = base pair

*no proxy SNP was identified for rs115124923 on chromosome 6; this SNP was not included in the final score calculation.

**Table S3. Within-Cohort Age of AD Onset by PGS Tertiles**

| Cohort | PGS Tertile | Estimated Mean | 95% CI Lower Bound | 95% CI Upper Bound |
| --- | --- | --- | --- | --- |
| ADNI | Low | 83.27 | 81.38 | 85.17 |
|  | Mid | 81.41 | 79.42 | 83.40 |
|  | High | 74.69 | 73.67 | 75.72 |
| LEADS | Low | 57.07 | 56.21 | 57.93 |
|  | Mid | 58.25 | 57.51 | 58.99 |
|  | High | 57.50 | 56.38 | 57.88 |

PGS = Polygenic score; CI = confidence interval

**Table S4. *APOE* e4 Carrier-Stratified Age of LOAD Onset by PGS Tertiles**

| Cohort | APOE e4 Carrier status | PGS Tertile | Estimated Mean | 95% CI Lower Bound | 95% CI Upper Bound |
| --- | --- | --- | --- | --- | --- |
| ADNI | Non-carrier | Low | 83.80 | 81.83 | 85.77 |
|  |  | Mid | 84.02 | 81.16 | 86.88 |
|  |  | High | 79.49 | 75.44 | 83.53 |
|  | Carrier | Low | 76.19 | 72.23 | 80.15 |
|  |  | Mid | 77.91 | 75.24 | 80.48 |
|  |  | High | 74.23 | 76.34 | 75.24 |

PGS = Polygenic score; CI = confidence interval

**Table S5. LEADS Cognitive Domains Adjustment**

| Domain | Model F | Model p-value | Variable | B | t | p-value |
| --- | --- | --- | --- | --- | --- | --- |
| Episodic Memory | 51.21 | <0.001 | Education (years) | -0.06 | -2.25 | 0.026 |
|  |  |  | Sex | 0.05 | 0.45 | 0.652 |
|  |  |  | Age | -0.06 | -4.79 | <0.001 |
|  |  |  | MMSE | 0.14 | 13.07 | <0.001 |
| Language | 6.78 | <0.001 | Education (years) | -0.22 | -1.71 | 0.089 |
|  |  |  | Sex | 0.16 | 0.28 | 0.780 |
|  |  |  | Age | -0.03 | -0.52 | 0.602 |
|  |  |  | MMSE | 0.29 | 5.10 | <0.001 |
| Processing Speed and Attention | 73.12 | <0.001 | Education (years) | -0.19 | -4.03 | <0.001 |
|  |  |  | Sex | 0.44 | 2.09 | 0.038 |
|  |  |  | Age | 0.01 | 0.23 | 0.816 |
|  |  |  | MMSE | 0.36 | 16.81 | <0.001 |
| Visuospatial Skills | 42.09 | <0.001 | Education (years) | -0.26 | -3.02 | 0.003 |
|  |  |  | Sex | -0.22 | -0.58 | 0.564 |
|  |  |  | Age | 0.05 | 1.15 | 0.251 |
|  |  |  | MMSE | 0.52 | 12.93 | <0.001 |
| Working Memory | 47.60 | <0.001 | Education (years) | -0.20 | -4.33 | <0.001 |
|  |  |  | Sex | 0.43 | 2.05 | 0.043 |
|  |  |  | Age | -0.05 | -2.44 | 0.016 |
|  |  |  | MMSE | 0.39 | 11.94 | <0.001 |

**Table S6. Pairwise Comparisons for Processing Speed and Attention Domain by PGS Tertiles**

| Domain | Pairwise Comparison | Test Statistic | Standard Error | p-value | Adjusted p-value |
| --- | --- | --- | --- | --- | --- |
| Processing Speed and Attention | Mid-Low | 16.34 | 9.76 | 0.094 | 0.283 |
|  | Mid-High | -26.10 | 9.19 | 0.005 | 0.014 |
|  | Low-High | -9.77 | 9.56 | 0.307 | 0.920 |
